# Navigating who I was and who I am online: How people with dementia use social media platforms to support identity

**DOI:** 10.1177/14713012241292659

**Published:** 2024-10-11

**Authors:** Catherine V Talbot, Daisy Roe, Melissa Brunner

**Affiliations:** Department of Psychology, 6657 Bournemouth University, UK; Ageing and Dementia Research Centre, 6657 Bournemouth University, UK; Department of Psychology, 6657 Bournemouth University, UK; Faculty of Medicine and Health, 4334 The University of Sydney, Australia

**Keywords:** alzheimer, online communities, support, self-disclosure, digital technology, affordances

## Abstract

A diagnosis of dementia can have a powerful impact on identity, and social media platforms offer promising avenues for identity expression and reconciliation. Addressing limited research in this area, we used semi-structured interviews to explore how 10 people with dementia used social media to navigate their identity. Our thematic analysis produced four themes, showing how social media platforms afford unique opportunities for self-expression, visibility, and association, thereby empowering users to maintain their sense of self, challenge stereotypes, and foster community connections. Additionally, social media facilitated a multifaceted and holistic sense of identity beyond the confines of diagnosis. While there were concerns about online self-disclosure, sharing experiences of dementia had therapeutic benefits, aiding in acceptance and adjustment. Participants also leveraged social media to establish continuity between their pre- and post-diagnostic selves, providing a sense of stability amid uncertainty. With the increasing prevalence of social media use among people with dementia, proactive measures by healthcare professionals, policymakers, technology developers, and carers are required to cultivate online experiences that are safe, supportive, and inclusive of people with dementia.

There are approximately 55 million people living with dementia worldwide, which is expected to reach 150 million by 2050 (GBD Dementia Forecasting Collaborators, 2022). Dementia follows a trajectory distinguished by three phases – early, middle, and late – with each presenting unique challenges. In the early stages, noticeable declines in instrumental daily activities occur, such as using the telephone, shopping, and household tasks (Pérès et al., 2008). These impairments can diminish independence, confidence, and the ability to ‘live well’ (Martyr et al., 2019). While many people with dementia demonstrate resilience and continue to experience joy and purpose following diagnosis (Wolverson et al., 2016), they also experience feelings of loss, depression, frustration, shame, and loneliness (Górska et al., 2018; Victor et al., 2020; Ward et al., 2022). Consequently, there is a pressing need to identify cost-effective, far-reaching methods to support people in living with and adapting to dementia.

Extensive research has focused on the impact of dementia on identity, emphasising its relative endurance throughout the disease trajectory (Caddell & Clare, 2010). However, a diagnosis of dementia can prompt shifts in identity, involving a ‘changing self’ marked by changes or losses in working, familial, and social roles (Greenwood & Smith, 2016; Griffin et al., 2016; Spreadbury & Kipps, 2019). This transitional phase also brings uncertainty about the future, requiring emotional readjustment, reconciliation of perceived losses, and re-evaluation of what lies ahead (Lishman et al., 2016; Scott, 2022; Yates et al., 2021). Identity is further impacted by stigma, which can be understood as a phenomenon in which an individual or a group is discredited by society due to an attribute (Goffman, 1963), culminating in diminished power in social relationships, isolation, fewer positive social interactions, and delayed support seeking (Phillipson et al., 2015; Piver et al., 2013; Ryan et al., 2021). This stigma is partly fuelled by media representations of dementia (Thomas & Milligan, 2018), portraying affected individuals as ‘sufferers’ and depicting symptoms in unrealistic, overly negative ways (Gerritsen et al., 2014; Peel, 2014). Additionally, advanced dementia dominates portrayals of the disease, thus exacerbating public stigma (Gove et al., 2016). Sabat (2003) theorised that this ‘malignant positioning’ hinders social opportunities and leads to withdrawal from meaningful social roles (Sabat, 2019; Sabat et al., 2011). This public-stigma can shape self-stigma, resulting in shame, reduced self-esteem, and a loss of confidence and withdrawal from daily activities (see Nguyen & Li, 2020). Therefore, people with dementia are not only disadvantaged by the disease, but also societal actions and attitudes.

Interventions have been developed to support self and identity among people with dementia (Caddell & Clare, 2011), with a growing emphasis on the potential of digital technologies (e.g., Goodall et al., 2021a; Sweeney et al., 2021). The functional online environments of social media, which foster user-generated content creation and sharing and include platforms like Facebook, Twitter, and online forums (Boyd & Ellison, 2007), offer valuable spaces for identity work, by facilitating self-expression and social connection. While research involving people with dementia is limited, extensive studies with other user groups demonstrate the value of online platforms for identity expression and exploration during periods of identity distress (e.g., Talbot et al., 2022; Thomas et al., 2017). Positive identity outcomes have been observed in studies with people with traumatic brain injury, who face similar communication difficulties to those with dementia. For example, Brunner et al. (2021) found participants appreciated the freedom of expression and control over self-presentation in social media spaces. Similarly, research with older adults – a demographic increasingly active on social media (Smith & Anderson, 2018) – has highlighted its supportive role in reconciling new identities. Blogging, for example, can support the continual refinement of identity, allowing reflection and negotiation of new roles such as a retiree or older person (Brewer & Piper, 2016). While dementia affects people of all ages, its prevalence increases with age (Baumgart et al., 2015), suggesting that people with dementia are part of this growing cohort engaging with social media.

Despite substantial research highlighting the benefits of social media for carers and family members of people with dementia (e.g., Anderson et al., 2017; Gkotsis et al., 2020), studies focusing on people with the diagnosis remains scarce. Initial research indicates that people with dementia use a variety of platforms, including Facebook (Craig & Strivens, 2016; Talbot et al., 2024), Twitter (e.g., Talbot et al., 2020a, 2020b; Thomas, 2017), online forums (Johnson et al., 2020; Talbot & Coulson, 2023), and blogs (Kannaley et al., 2019). Recent findings suggest that Facebook is the most prevalent platform among this demographic, and people with young-onset dementia are more likely to use social media than those with late-onset dementia (Kohl et al., 2023; Talbot et al., 2024). Most studies have highlighted social media’s role in enabling disclosure of lived experiences (e.g., Kannaley et al., 2019; Talbot & Coulson, 2023), enabling users to define aspects of their identities post-diagnosis (Talbot et al., 2021). However, research has predominantly focused on *general* use of specific social media platforms rather than explicitly examining online activities in relation to identity. A notable exception is Talbot et al.’s (2021) interview study, finding that Twitter facilitated a re-shaping of identity by providing access to self-relevant information and fostering connections within the dementia community. Moreover, existing evidence primarily focuses on individual platforms rather than the wider social media ecosystem (see Buss et al., 2022), lacking understanding of how various platforms facilitate identity-related processes. We address this gap in our study in which we explored how people with dementia use social media in relation to identity.

## Method

This qualitative study employed an iterative, reflexive thematic analysis approach (Braun & Clarke, 2019) and was part of a larger research project exploring online self-identity of people with neurological conditions. Ethical approval was obtained from Bournemouth University [Project 33976] and the University of Sydney [Project 2021/013] prior to study commencement.

### Recruitment

Purposeful sampling was employed to recruit adults with dementia, meeting the following inclusion criteria: aged 18 years or older; had a self-reported diagnosis of dementia; used at least one social media platform; able to participate in a remote interview discussion in English; and capacity to provide informed consent. Recruitment posts were sent out via dementia support groups and social media, with links to an online REDCap survey containing the Participant Information Statement and Consent Form. Researchers then contacted respondents via email to schedule remote interviews and collect initial information regarding the social media platform and posts they intended to discuss. Before interviews commenced, informed consent was confirmed using a study-specific “Assessment of Capacity to Consent” questionnaire (Jeste et al., 2007), which assessed understanding, retention, ability to weigh-up information, and communicate a clear and informed decision.

### Participants

Ten people participated in this study, comprising three female and seven male participants. Their ages ranged from 51 to 72 years and they resided in various locations: United Kingdom (*n* = 6), Republic of Ireland (*n* = 1), Canada (*n* = 2), and Australia (*n* = 1). Table 1 summarises participants’ characteristics.

**Table 1.** Participant characteristics.

Pseudonym	Gender	Age	Time since diagnosis	Social media used and discussed
Mark	Male	68	45 days	Facebook, twitter, online forums
Joseph	Male	63	5 years	Facebook, twitter
Bethany	Female	56	2 years	Facebook, twitter, blogs
Ralph	Male	51	10 years	Twitter, facebook, instagram
Linda	Female	65	9 years	Facebook, twitter, blogs
Andy	Male	57	4 years	Facebook, twitter
Bryan	Male	72	14 years	Twitter
Charlotte	Female	58	4 years	Facebook, twitter
Stuart	Male	64	8 years	Facebook, twitter
Lee	Male	62	14 years	Twitter

*Note: Although ‘Twitter’ is now called ‘X’, during the interviews this name change had not occurred. Therefore, the platform is referred to as Twitter throughout this paper.

### Data collection

Interviews were conducted between March 2021 and May 2022, using a photo-elicitation approach (Wang & Burris, 1997) and modified scroll-back method (Robards & Lincoln, 2017). Prior to the interviews, participants shared the handle of a chosen social media platform along with four-six social media posts showcasing their experiences, values, and identity. Participants shared their social media content either by directly sending posts to the researchers (via screenshots or links) or by providing their username, allowing the researchers to scroll through their profile during the interview. Interviews were conducted via Zoom, telephone, or email, depending on personal preference (CVT; MB). A semi-structured conversational style interview style was used, guided by a predetermined topic guide designed to enable participants to tell their own social media stories and contemplate their online personas (Patton, 2014). Additional tailored questions were incorporated to focus on individual experiences, using social media visuals as reference points. Researcher field notes were documented during and after the interviews to capture initial reflections. Verbatim transcriptions were generated from recordings (DR) using Microsoft Word for subsequent analysis.

### Data analysis

We used a reflexive thematic analysis approach (Braun & Clarke, 2019) to examine shared experiences across the participants. The first and second authors (CVT; DR) immersed themselves in the data by first reading and re-reading transcripts, documenting initial coding ideas. Subsequently, these authors had initial discussions to unpack data observations, with theme development guided by an interpretivist lens. Initial coding was performed by CVT and DR, with the resulting codes mapped into initial themes using Miro, an online platform with a virtual whiteboard feature. Definitions for initial themes were then produced, and subsequently reviewed and refined by MB. Final themes were iteratively generated by CVT, with input from all co-authors. To ensure findings remained firmly grounded in the data, rich descriptive summaries of the themes were created with theme names drawn directly from participant quotations.

## Results

Our thematic analysis produced four themes illustrating how people with dementia used social media in relation to their identity: (1) Self-disclosing dementia; (2) Sustaining identity; (3) I’m more than my diagnosis; and (4) Platform identity affordances. These themes highlight the ways in which people with dementia use social media to construct, negotiate, and express their identities, while also demonstrating the significant role of platform affordances in shaping identity expression (see Table 2).

**Table 2.** Themes overview.

Theme name	Definition
Self-disclosing dementia	People with dementia deliberated on whether to disclose or conceal their diagnosis on social media. Those who chose to share their diagnosis tried to provide a nuanced portrayal of dementia, including positive and negative aspects.
Sustaining identity	Social media allowed people with dementia to maintain aspects of their identity, facilitating a sense of continuity between their pre and post diagnostic selves.
I’m more than my diagnosis	Engaging with social media enabled people with dementia to express aspects of themselves beyond their dementia diagnosis, facilitating a more holistic and multifaceted sense of self.
Platform identity affordances	Social media platforms provided identity affordances of self-expression, visibility, and association.

### “It’s showing the world”: Self-disclosing dementia

When participants engaged with social media, a key consideration was whether to disclose or conceal their dementia diagnosis. This decision involved careful deliberation and was often shaped by stigma, particularly a fear that disclosure might alter how others perceive them. For example, one participant explained how they initially refrained from discussing dementia online to avoid malignant positioning and resist stereotypes commonly associated with the diagnosis:

I can see myself posting more about dementia in the future. But at the same time, I still want people to see me as someone my age, who still is interested in life (Ralph)

The decision to disclose a dementia diagnosis was also context-dependent, depending on the specific platform and audience. Participants explained how designated Facebook groups and online forums for people with dementia created environments conducive to self-disclosure. These spaces fostered a shared understanding among users, creating comfort and openness in discussing experiences.

I put much more detailed stuff, the Alzheimer’s Society have a forum page, and I use that to describe my journey in more detail than I put on Twitter… Because it’s a forum of people whose interest is in this condition and who you don’t necessarily have to explain what a SPECT scan is because many people will know about that (Mark)

Some participants purposefully disclosed their diagnostic identity in public social media platforms like Twitter and blogs to educate others about the realities of dementia, aiming to foster a more nuanced and empathetic understanding among the general public, organisations, researchers, policymakers, and friends and family of people with dementia. Participants recalled instances where such self-disclosure positively impacted others. One participant discussed the effects within their local community, which inspired others to disclose their own experiences and fostered social support:

There’s another man in the village who everybody knows has dementia, but the family didn’t announce it because they were too embarrassed. But when they saw how kind people were being to me on the Facebook page, they suddenly announced it... And now they’re being helped because people look out for him and help him (Linda)

Disclosing a diagnosis of dementia also had personal benefits for participants, particularly when expressed through blogs and micro-blogs. Writing about their experiences provided an important outlet for therapeutic self-expression, aiding understanding and acceptance of the realities of living with dementia.

It’s a way of me thinking through, this is an odd thing that is happening. Because by sharing it helps me come to terms with it. (Mark)

I’ve got so much going round in my head, when I put it to paper, it’s like a release. I suppose it’s like if you go to a counsellor (Bethany)

Most participants intentionally sought to communicate positive aspects of their lives through public social media channels. They explained the value of intentional self-disclosure in actively combatting negative stereotypes and steering prevailing narratives towards a more balanced perception of dementia. Participants believed this approach could inspire hope among others affected by dementia, by affirming that they can lead fulfilling lives despite the challenges posed by the diagnosis.

We know my memory will deteriorate. It’s letting people know that, but it’s not going to just deteriorate like I got my diagnosis and then tomorrow I’m going to be sat in a chair. So, I wanted to take them on my journey showing them that you can live and enjoy your life (Bethany)

However, participants were acutely aware of the importance of avoiding an unrealistically positive portrayal of dementia. Instead, their intention was to offer a balanced and authentic representation that included both positive and challenging aspects of their lives. This approach to self-disclosure facilitated support within online communities and helped normalise experiences of dementia, promoting open conversations and motivating others to seek the support they may need.

If I tweet during the night while I'm in the middle of a horrific dream, and that is real time… It helps people, and it makes people feel like it's okay talk about, it's okay to be this way (Andy)

Online self-disclosure was influenced by individual motivations, stigma, and context. By sharing their experiences authentically and intentionally, participants aimed to challenge stereotypes, foster understanding, and promote support within online networks, contributing to a more inclusive and nuanced discourse surrounding dementia.

### “A continuation of who I was”: Sustaining identity

Most participants reported experiencing losses or changes in their identities following diagnosis. However, social media provided participants with a valuable means of preserving and remaining connected to certain aspects of themselves. Specifically, social media facilitated a sense of continuity between pre- and post-diagnostic selves, offering a comforting sense of stability amid a time of uncertainty.

It’s a continuation of who I once was… And a lot of who I once was hasn’t continued. I’m living in a different place, my circumstances have completely changed. But this is still me. And that’s important to me. (Mark)

Discussions of identity continuity through social media often centred around participants’ working identities. Despite all participants having retired from paid employment, they found ways of sustaining their professional identities through social media by leveraging the skills and expertise acquired throughout their careers. One participant described this as a “transfer of skills”, drawing parallels between his online dementia advocacy work and prior work in policymaking.

You don’t make the point by slamming your fist on the desk and do it or whatever. You know, it’s just keep plugging away and chipping away at something and you make the point and that’s how I did it at the airline and that’s what I do now. (Bryan)

Similarly, other participants shared how they used the skills honed during their professional careers to support others affected by dementia via social media. By applying these skills, participants became valuable sources of practical information within online communities, providing information about the support available to people with dementia and demystifying complex processes such as medical tests. While participants engaged in these activities to help others, they also personally derived a sense of purpose from sharing knowledge and guidance.

I deliberately shared those details, because you go online, and nobody tells you about that. It’s sort of vague, you know, you’ll be injected with an isotope, nobody explains about the nurse creeping in on tip toe, that’s all a bit peculiar. Erm, so it’s a bit of, sort of, bit of journalism I suppose. (Mark)

Finally, check if they are receiving all the State Benefits applicable, I used to be a Benefit Fraud Manager so know the system, how to complete forms correctly (Joseph)

Another key way in which social media sustained identity was through the preservation of memories, as evidenced by one participant who noted the utility of these platforms as a memory repository. For this participant, social media provided a dedicated space to capture and store their experiences. Additionally, it enabled them to share these memories with family members, overcoming challenges associated with recalling self-relevant information.

For me, to know what I’ve been doing. And for my daughters to know what I’ve been doing. Because I forget to tell them, but they can just look at my blog. (Bethany)

For these participants, social media played a significant role in sustaining aspects of identity during a time of uncertainty, by fostering identity continuity through leveraging skills and expertise, and preserving memories.

### “The dementia doesn’t define who I am”: I’m more than my diagnosis

Participants found that social media enabled them to explore aspects of themselves beyond their dementia diagnosis, thus facilitating a more holistic and multifaceted sense of self. In offline interactions, participants often felt reduced to their diagnosis. As stated by one participant, “they see the dementia, they don’t see you” (Bethany). In contrast, social media provided a space where participants could freely communicate and explore other aspects of themselves, without the constraints of their diagnostic identity and associated stereotypes. One participant with young-onset dementia, for example, described using Twitter to engage with various interests, thus rejecting stereotypes of old age, strengthening identification with their age group, and resisting being exclusively defined by the diagnosis.

I feel very old when I tell people that I have dementia. And to post things like the UFC, or different things, it makes me feel like I’m in my age group again…. Because the dementia doesn’t define who I am as a person, and it doesn’t define my interests that I like to follow (Ralph)

Participants consistently expressed a desire to be seen as complete individuals, with diverse interests and multiple identities. Social media platforms were instrumental in communicating these aspects of identity, without being constrained by the diagnosis. For example, one participant recalled sharing their photography in a local Facebook group, foregrounding their hobby over their diagnosis:

And people who didn’t know me then began to call me the camera lady instead of Linda with dementia…And now they all realise I have dementia, but I’m still Linda the camera lady first (Linda)

Importantly, participants’ experiences highlight how social media platforms are spaces where different aspects of identity intersect, including diagnostic identities and other personal interests. Another important aspect of identity discussed by some participants was creativity, and the role of social media in providing outlets for creative expression through art, photography, crafting, or other creative projects. For these participants, social media platforms enabled them to continue to embrace and showcase their creativity, while also educating and inspiring others through their creative expressions.

I put little videos out so that you can replay them, you know, as many times as you want. So yes, so the crafts went down really well. And people were saying wow what an inspiration, I didn’t realise that we could still do these sorts of things… I’ve always, always done crafting.… But now it might take me longer to do things but it’s the achievement at the end of it (Bethany)

For the participants in this study, social media played a pivotal role in facilitating a holistic and multifaceted sense of self beyond the constraints of a dementia diagnosis. This highlights the empowering potential of social media in enabling people with dementia to assert their identity and actively engage with interests that bring joy, purpose, and a sense of fulfilment.

### “It’s a nice way of communicating”: Platform identity affordances

Social media platforms were described as having distinct affordances that enabled identity work, particularly self-expression, visibility, and association affordances (see Kitzie, 2019). They acknowledged how social media platforms afforded unique opportunities for self-expression, enabling them to overcome offline communication challenges by empowering them to communicate in a textual format. For example, one participant described difficulties with word recall in offline conversations but found that typing their thoughts online provided a simpler and essential “escape from dementia”.

Because I can’t think of the words quick enough. Which is why I use typing, because I can think and type quicker than I can speak. (Linda)

Participants also valued the multimodal communication facilitated by social media platforms, including text, images, emojis, and videos. The multimodal nature of communication provided multiple avenues for self-expression, allowing participants to effectively convey their thoughts, emotions, and experiences in diverse ways, thus amplifying their capacity to communicate their identities and engage with others.

Now I think I put on more photographs rather than text on a lot of them because it’s a way of telling my story and I can just have little snippets in. (Bethany)

When discussing the self-expression affordances of social media, participants tended to express a preference for Twitter. They specifically noted how the “concise” and “short snappy” nature of microblogs streamlined communication and reduced cognitive demands, which was generally more accommodating of their communication needs than other platforms.

It’s a nice way of communicating. Short sentences. (Andy)

They were restricted in the number of words they could put in a sentence, so that enabled me to be able to still read because I can’t read long things anymore. (Linda)

Another affordance noted by participants was related to visibility, whereby more public social media platforms such as Twitter provided a powerful means of amplifying their experiences and perspectives to a broad audience. Those engaged in dementia advocacy perceived this visibility as pivotal in their roles as dementia activists. Social media provided them with a ‘stage’ to raise awareness and influence research, practices, and policies, having far-reaching potential to make an impact.

I have a friend and he knew that I was interested in having my voice heard, you know. And he says, have you heard of Twitter? I’ve heard of it, I’ve never used it. He said, you should really sign up for Twitter. And he explained it carefully and he even helped me set up an account. And then at that point I started learning. Wow. You know, media is frigging powerful. (Stuart)

In addition to these affordances, participants also noted the association affordances of social media, referring to the facilitation of social connections beyond direct networks. Participants particularly valued how social media enabled connections between people with dementia, providing an important source of community identity and access to social resources to cope with the diagnosis, particularly for those living rurally.

Without Twitter, I would feel alone with dementia as I live very rurally (Joeseph)

It’s just encouraging one another and it’s all coming together and yeah, it just feels like a little community. (Bethany)

However, the association and visibility affordances of social media also presented risks. One participant discussed association-related challenges in Facebook groups, where they were repeatedly exposed to negative experiences of dementia, which they did not identify with and found distressing. A few other participants mentioned encountering hostility from other users due to not presenting in a stereotypical manner, with one participant stating that he needed “a bit of a thick skin” (Ralph). This issue was demoralising and upsetting for participants and was explicitly discussed in relation to Twitter, where participants could disclose positive experiences of dementia with a broad audience.

Twitter can be very aggressive against you sometimes because you can, because they only see the side of me that can type. So, they question the dementia and question the reality… One woman she just left Twitter because she couldn’t cope with the constant barrage (Linda)

Social media platforms appear to offer unique affordances that facilitate identity work for people living with dementia, in ways that may not be readily available offline. However, these affordances also present risks, such as repeated exposure to distressing negative experiences and encountering hostility from other users.

## Discussion

In this study, we explored the ways in which people with dementia use social media platforms to navigate their identities. Our findings highlight the value of social media in exploring, maintaining, and reshaping their sense of identity following diagnosis. Central to these experiences was the decision about whether to disclose their diagnosis online, weighing the potential stigma against the benefits of social connection and advocacy. Writing about personal experiences on social media provided important avenues for therapeutic self-expression, challenging stereotypes, and presenting a nuanced representation of life with dementia. Social media platforms also served as spaces to maintain identities beyond a dementia diagnosis, thereby fostering a more holistic sense of self. By leveraging professional skills and preserving memories, people with dementia were also able to sustain various aspects of their identity. The unique identity affordances of self-expression, visibility, and association offered by social media platforms became invaluable sources of empowerment and post-diagnostic support for people with dementia.

The decision to disclose a dementia diagnosis on social media was complex and deeply personal, often shaped by stigma and intended audience. Our findings align with Kohl et al. (2023), who also observed that public self-disclosure aimed to challenge misconceptions and raise awareness, highlighting the value of public social media platforms for dementia advocacy and education. While many participants in our study openly disclosed their diagnosis, others may choose to conceal or selectively manage their diagnostic identities (Thorsen et al., 2020; Weaks et al., 2015). In such cases, users may prefer online spaces offering heightened anonymity and connection to others with similar experiences (e.g., Talbot & Coulson, 2023). Similarly, research on other stigmatised contexts has shown initial preferences for platforms providing anonymity and network separation during personal disclosures (e.g., Andalibi, 2019; Haimson, 2018; Talbot et al., 2022). People with dementia may follow a similar trajectory to others with stigmatised identities, gradually disclosing their diagnostic identity within less anonymous social media contexts, leveraging close social ties for support and widespread disclosure (e.g., Fox & Ralston, 2016; Owens, 2017). Further research is needed to understand attitudes towards such disclosures online, including how self-disclosure evolves over time, to inform interventions. Collaboration between technology designers and people with lived experience, for example, could produce features enhancing privacy and visibility of disclosures, such as audience segmentation and selective sharing, thus empowering users to disclose their diagnostic identity safely and comfortably.

Writing about experiences of dementia on social media, particularly through blogs and micro-blogs, provided a therapeutic outlet that aided understanding and acceptance of life with dementia. This mirrors recent research on bloggers with dementia, which highlights the personal benefits of storytelling for finding one’s voice and exercising autonomy and control over narratives (e.g., Brooks & Savitch, 2022). The therapeutic benefits of writing as a tool for self-discovery, healing, and reconciliation have been well-documented, offering opportunities for personal growth and recovery of social roles (e.g., Ryan, 2006; Ryan et al., 2005). Writing does not demand perfect grammar, thus providing useful opportunities for self-expression for people with dementia (Ryan et al., 2009). Similarly, our findings suggest digital storytelling via social media platforms may help overcome challenges encountered in offline communication, such as word-finding difficulties. The increasing accessibility and flexibility of online spaces, such as blogging platforms, makes them potentially well-suited for people with dementia, similar to bloggers with other well-researched chronic health conditions (e.g., Murphy et al., 2020; Tsai et al., 2018). These platforms offer a versatile space for self-expression, facilitated by accessibility features such as voice-to-text technology (Brooks & Savitch, 2022). Future research could explore the efficacy and scalability of digital storytelling interventions for people with dementia, including longitudinal studies to understand the long-term benefits and challenges as dementia progresses. Healthcare professionals and carers could also consider integrating digital storytelling into dementia care plans. Additionally, exposure to digital narratives of people navigating life with dementia may provide a source of hope and encouragement to those recently diagnosed, by evidencing the possibilities of living a fulfilling life despite the diagnosis.

Social media platforms enabled the participants in our study to explore aspects of themselves beyond their diagnosis, fostering a more holistic sense of identity without the constraints limiting offline interactions (Patterson et al., 2018; Sabat, 2019). This aligns with research emphasising the importance of maintaining diverse aspects of identity. For example, people with dementia have expressed a desire to be recognised as complete individuals, advocating to be acknowledged as ‘more than dementia’ (Beard et al., 2009). Additionally, engaging with personal interests can contribute to a sustained sense of self (Phinney, Chaudhury, O’Conner, 2007). Therefore, social media platforms offer promising opportunities to enrich the lives of people with dementia by enhancing wellbeing, fulfilment, and a sustained and multifaceted sense of self. Despite this, digital technologies for people with dementia are often overly paternalistic, failing to recognise personhood or support potentialities, abilities, and identities (see Lazar et al., 2017). We therefore recommend that social technologies are designed to support the higher-level needs of people with dementia, which includes facilitating a multi-faceted sense of identity.

In our study, social media was a valuable tool for sustaining aspects of identity following diagnosis, providing an important sense of continuity between pre- and post-diagnosis selves. Previous longitudinal research suggests people with dementia engage in processes of meaning-making and adaptation to cognitive decline, seeking an equilibrium between maintaining past identities and adapting to new ones (Steeman et al., 2013), with our research indicating social media platforms may serve as valuable spaces for identity continuity. Social media platforms offer unique opportunities for this, providing spaces for memory preservation, meaningful activities, and connections with others. By using platforms like Facebook, people with dementia can reminisce about past events using the memories function and participate in online communities that reflect aspects of their identity pre-diagnosis, and may be impacted by the symptoms of dementia. Moreover, research highlighting a link between autobiographical memory and identity (see Caddell & Clare, 2010), highlights the potential value of incorporating social media content into reminiscence therapy. Through curated photo albums, videos, and posts, users may be able to revisit memories, reinforce their sense of self, and preserve their personal narrative over time.

Social media platforms have been extensively studied for their identity affordances among stigmatised groups (e.g., Kitzie et al., 2018, 2019), but this perspective had not been applied to dementia prior to our study. Our research demonstrates that social media provides distinct identity affordances that facilitate self-expression, visibility, and association, thus offering opportunities not always available offline. The concise self-expression affordances of Twitter allowed people with dementia to overcome communication barriers associated with concentration and speech, mirroring research with other groups experiencing communication difficulties (Brunner et al., 2015; Hemsley et al., 2015). Additionally, our findings suggest that public-facing platforms like Twitter can amplify the voices of people with dementia for advocacy. Platforms such as Facebook and online forums provided association affordances, fostering connections and community engagement that may not be accessible offline, especially for those with rarer forms of dementia (Cations et al., 2017; Willis et al., 2018). Encouraging people with dementia to engage with online communities could alleviate isolation, promote identity, and aid adjustment to dementia. Clinicians may consider directing people with dementia to these online spaces for empowerment and social support. However, people with dementia must also be aware of risks, such as repeated exposure to negative experiences of dementia and the potential for encountering hostility from other users, particularly ‘dementia doubters’ (see Talbot et al., 2021). Future implementation of safeguarding measures and providing education on safe online practices are crucial to mitigate these risks. Future research could also explore additional affordances of social media for people with dementia by drawing upon affordances literature from other stigmatised contexts.

Our work coincides with the growing digitalisation of dementia care (Giebel, 2023). However, we acknowledge that not all people with dementia have the access, capability, or interest to engage with social media. Previous studies have highlighted some of the challenges people with dementia and their carers face, including difficulties with memory recall, concentration, typing, password management, as well as concerns about misinformation, malicious links, and scams (Piper et al., 2016; Talbot et al., 2021, 2024). It is also essential that our findings do not overshadow the urgent need to invest in offline services, particularly those which facilitate identity formation. However, the rising adoption of social media usage among younger generations (Smith & Anderson, 2018) suggests a rapidly shifting landscape in dementia care. Consequently, services, carers, policymakers, and technology developers must anticipate and prepare for the growing significance of social media in dementia care. This may involve a concerted effort to bolster digital access and literacy within relevant services (see Hicks et al., 2023). Designers of social media must also focus on creating online spaces which are more inclusive of people with dementia, to facilitate supportive and safe online experiences.

### Limitations and future directions

While our study provides valuable insights into the experiences of people with dementia in using social media to shape their identities, there are some limitations. Firstly, our study focused on people with dementia who actively use social media platforms, meaning the experiences of those who have stopped using social media or have never used it remain unexplored. It is now critical for researchers to work with non-users of social media to better understand the barriers they face. Additionally, people with more severe cognitive impairments or limited digital literacy skills were not represented in our sample, potentially skewing our understanding of the role of social media’s impact on identity. While social media might not appeal to everyone within this demographic, ensuring the accessibility and inclusivity of online spaces remains crucial for upholding the wellbeing and social inclusion of people with dementia.

Furthermore, our research primarily focused on personal identity, rather than social identity. Recent research has highlighted the value of adopting social identity frameworks to understand engagement with social technologies (e.g., Stuart et al., 2022). Future studies could benefit from integrating such frameworks to gain a deeper understanding of how social media shapes identity among people with dementia. Additionally, the cross-sectional nature of the study limits our ability to capture the how social media usage influences identity over time. In longitudinal research focused on terminal cancer, Taylor and Pagliari (2018) found changes in Twitter content across the disease trajectory, with later tweets focusing more intensely on social and caregiving support. Given the progressive nature of dementia, it is plausible that social media engagement and its impact on identity similarly evolves throughout the disease progression. Furthermore, our study did not explore the intersectionality of participants’ experiences of identity, such as gender, sexual identity, and ethnicity, which may intersect with dementia to shape online experiences. For example, LGBTQ + people with dementia face distinct challenges, including a lack of support, dual stigma, and gaps in service provision (Candrian et al., 2023; Nowaskie & Sewell, 2021). Consequently, they may use social media to support their identities in ways different from what we have outlined in this paper. Future research should explore these dimensions to gain a deeper understanding of how various identities influence social media use among people with dementia.

Finally, the dynamic nature of social media and digital technologies means that our findings may become outdated over time as platforms evolve and new features are introduced. Continuous monitoring and research are necessary to keep pace with these changes and ensure that digital interventions remain relevant and effective for people with dementia. Future research endeavours we identified throughout this paper provide an initial roadmap for research in this space. That is, research exploring the efficacy and scalability of digital storytelling and reminiscence therapy interventions for people with dementia; and building our understanding of the longitudinal benefits and challenges of social media use and social identity changes as dementia progresses.

### Conclusion

In conclusion, our study highlights the valuable role of social media platforms in shaping the identities of people living with dementia. By affording self-expression, visibility, and association, social media can empower people with dementia to maintain a sense of self, challenge stereotypes, and connect with others affected by dementia. Importantly, these platforms offer a dynamic space for people with dementia to explore aspects of identity beyond diagnosis, contributing to a more holistic sense of self. While there were concerns about online self-disclosure, writing about personal experiences of dementia provided therapeutic benefits. People with dementia also used social media to experience a sense of identity continuity between their pre- and post-diagnostic selves, by preserving memories and leveraging professional skills, which offered a sense of stability amid uncertainty. As social media usage among people with dementia continues to rise, it is essential that healthcare professionals, policymakers, technology developers, and carers proactively prepare for this trend. Specifically, they must work collaboratively to cultivate online experiences that prioritise wellbeing, social support, safety, and social inclusion for people with dementia.
